# Clival metastasis of hepatocellular carcinoma: a case report of a rare skull-base malignancy

**DOI:** 10.3389/fonc.2025.1567187

**Published:** 2026-01-12

**Authors:** Tomislav Felbabić, Roman Bošnjak, Jure Urbančič, Matic Bošnjak, Domen Vozel

**Affiliations:** 1 Department of Neurosurgery, University Medical Centre Ljubljana, Ljubljana, Slovenia; 2 Faculty of Medicine, University of Ljubljana, Ljubljana, Slovenia; 3 Department of Otorhinolaryngology and Cervicofacial Surgery, University Medical Centre Ljubljana, Ljubljana, Slovenia; 4 Faculty of Medicine, Institute of Pathology, University of Ljubljana, Ljubljana, Slovenia

**Keywords:** hepatocellular carcinoma, clivus, metastasis, endoscopic endonasal surgery, case report

## Abstract

**Introduction:**

Hepatocellular carcinoma is the most common neoplasm of the liver. It metastasizes mainly to local lymph nodes, lungs, adrenal glands, vertebrae, pelvis, ribs and long bones. Metastasis to the clivus is extremely rare. Only a few cases have been described in the literature. Therefore, there is currently no clear consensus on the optimal treatment: biopsy only or maximum safe removal.

**Case report:**

We present a 68-year-old lady who presented with dysfunction of the 6th and 12th cranial nerves. Imaging showed tumor formation in the clivus, most likely a metastasis. Further diagnostics revealed no clear primary tumor. The patient therefore underwent surgery for local decompression and simultaneous biopsy for pathohistologic examinations. These showed that the most likely metastasis was a hepatocellular carcinoma. Subsequent diagnostics revealed highly elevated alpha-feto protein levels and liver MR revealed a suspected multifocal hepatocellular carcinoma only after correlation with the pathohistologic findings. One month after surgery, the condition suddenly worsened due to severe local recurrence and hemorrhage. This was followed by palliative oncological treatment with whole head irradiation.

**Conclusion:**

In the case of a suspected clivus metastasis of unknown origin, it is reasonable to take tumor markers. This increases the likelihood of an appropriate diagnosis and avoids unnecessary and risky surgery. If all tests are inconclusive, a biopsy of the lesion is the diagnostic gold standard. The treatment of metastatic disease in the clivus (in our case a hepatocellular carcinoma) remains palliative.

## 1 Introduction

Hepatocellular carcinoma (HCC) is the most common primary liver cancer, the sixth most common malignancy and the third most common cause of death in cancer patients (1). It occurs most frequently in patients with chronic liver disease (2) and in about 20% of cases in patients without previous liver disease (3). It mainly metastasizes to regional lymph nodes, distally also to lungs, adrenal glands and bones (4). The most common skeletal invasion locations are spine, pelvis, long bones and ribs. Skull infiltration is very rare and not many articles have been published. They are most often in the form of case reports (5–11). The most comprehensive review of the literature was conducted by Guo et al. who found a total of 59 cranial metastases from HCC (12). Out of those 59, 31 were in calvaria, 16 in skull base and 12 in facial bones. However, the 16 skull base cases were not further identified, so it is unknown, how many of those were found in clival area. Overall incidence of HCC skull metastases was estimated at 0,4% -1,6%. Because of the anatomical relationship of clivus with cranial nerves, cranial nerve dysfunction is usually the first symptom of the metastasis (13). Abducens palsy is the most common (14). They can also be asymptomatic incidental finding, detected in the course of radiological staging of the primary disease via brain CT or MR scan. Since these lesions are rare, there is yet no clear consensus about the best line of treatment; just a biopsy or maximal safe resection (15). In this article, we present the case of a patient with a clival metastasis as the first manifestation of previously undiagnosed HCC.

## 2 Case presentation

The case report is prepared according to the CARE guidelines.

### 2.1 Initial presentation

**Figure 1:**
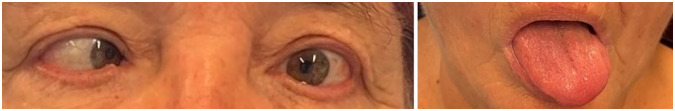
The photograph of the patient with a clival metastasis revealing left sided abducens and hypoglossal nerve palsy.

A 68-year-old woman with arterial hypertension presented to the emergency neurology service with a three-week history of headache and sudden onset of double vision. She denied excessive alcohol consumption. Neurological examination revealed left abducens and left hypoglossal nerve dysfunction (**Figure 1**). In addition, she reported an occasional dysphagia. The motor and somatosensory innervation of the face were normal.

### 2.2 Imaging studies

**Figure 2:**
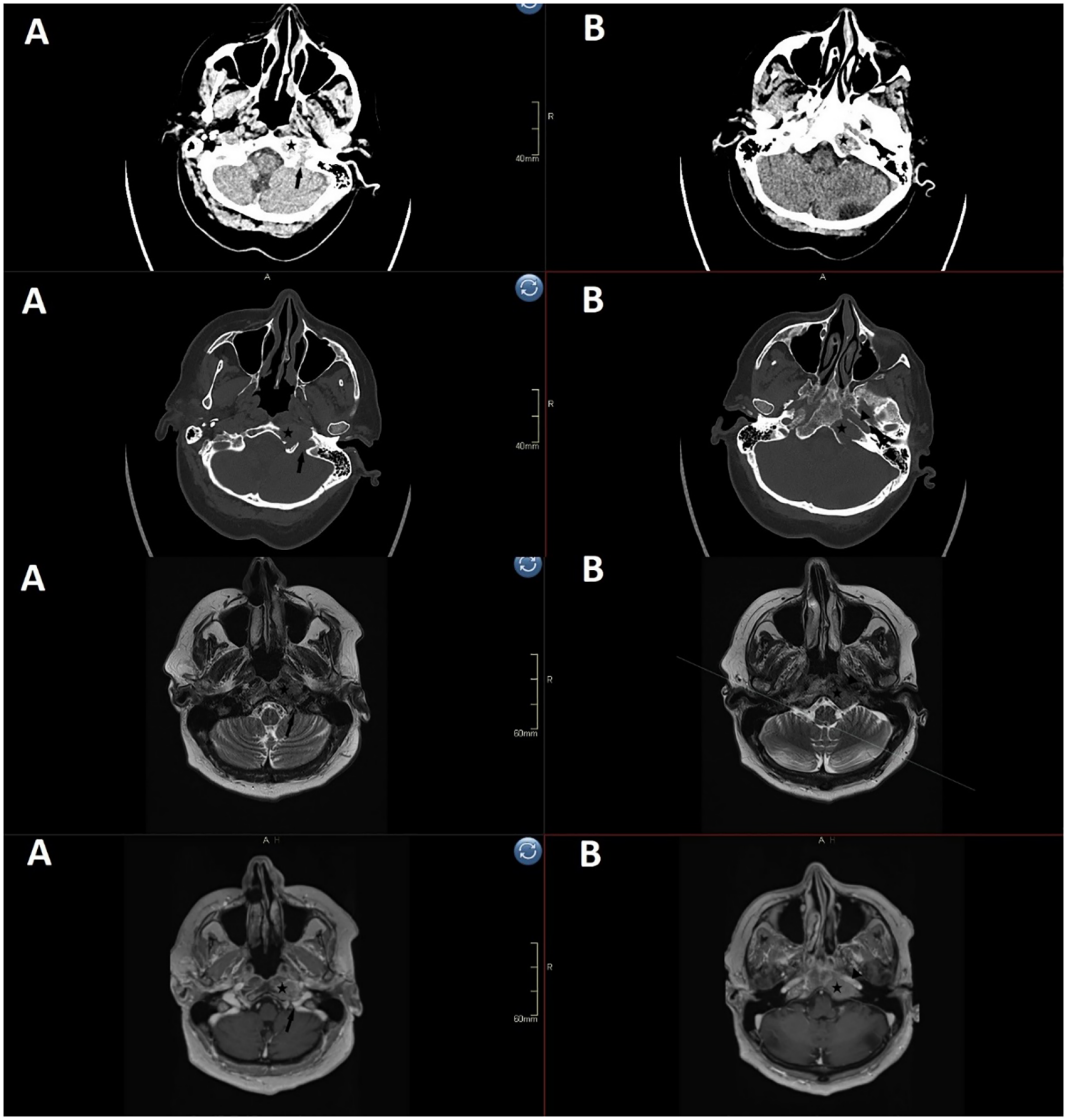
Preoperative imaging (1st row axial CT soft tissue window, 2nd row axial CT bone window, 3rd row axial T2 sequence, 4th row axial contrast-enhanced T1 sequence) shows a 2.4 x 2 x 1.5 cm erosion in the clivus and left petrous apex. The left condyle is intact, but the tumor invades the hypoglossal canal and extends to the jugular foramen **(A)**, erodes most of the lateral clivus from the petrosal part of carotid artery to the jugular tubercle **(B)**, and extends posterior to the paraclival carotid and petroclival fissure to via medial petrous apex toward the internal auditory canal. *(star: tumor, arrow: jugular foramen, triangle: petrosal part of carotid)*.

Head CT showed a soft tissue formation on the left lateral mass of the occipital bone, in close contact with the jugular foramen, measuring 2.4 x 2 x 1.5 cm. A contrast-enhanced head MRI was recommended, where radiologist described an expansive formation involving and destroying the left part of the clivus, including the left petroclival fissure. The tumor extended to the tip of the petrous apex and measured 2.7 x 1.8 cm in the axial plane and 3 cm craniocaudally. The tumor extended cranially to the internal auditory canal, medially to the midline of the clivus and laterally to the carotid and jugular foramen. It extended caudally into the pharyngeal recess where it partially eroded the left hypoglossal canal. The differential diagnosis was suspicious for a metastasis, chordoma, chondrosarcoma or a plasmacytoma (**Figure 2**).

After the imaging, the patient was examined at the neurosurgical outpatient clinic. Further imaging was recommended: MRI of the spinal canal and contrast-enhanced CT of the craniocervical junction, thorax and abdomen. None of the modalities identified a distant origin of clival lesion. On CT of the thorax, the radiologist described a 7 mm nonspecific nodular lesion in the mediastinal lobe of the right lung adjacent to the lesser subpleural fissure, without osteolytic or osteosclerotic changes. CT of the abdomen showed a normal-sized liver without pathologic focal lesions, no pathologically enlarged retroperitoneal, iliac or inguinal lymph nodes and a 4.3 cm hypodense endometrial lesion, for which the radiologist also recommended a pelvic MRI to differentiate the lesion as a submucosal myoma. All of the imaging studies were done outside our tertiary center. They were performed within 3 weeks of the initial neurosurgical examination. After review of the patient’s records in a multidisciplinary skull base pathology meeting, surgical decompression and safe expanded biopsy via an endoscopic endonasal transclival-transjugular approach was indicated (16).

### 2.3 Surgery

Surgical approach was endoscopic endonasal. Tumor removal was done via the left nostril using a 0° endoscope and a 3-hand technique.

An extended transrostral sphenoidotomy exposed the sellar floor, paraclival carotids, and lower clivus. The nasopharyngeal musculature was desinserted, exposing the anterior foramen magnum and C1 lamina. The pharyngeal tubercle was used as a landmark. Pulsations indicated proximity of the parapharyngeal carotid, though it wasn’t visualized.

A brownish, gelatinous extracranial tumor was resected. The frozen section showed a malignant epithelioid neoplasm, so chances for a chordoma or plasmacytoma were unlikely. Bone over the supracondylar cavity was drilled to access the intraclival tumor. Drilling extended from the sellar floor to the foramen magnum (FOM) and into the left lateral clivus, thinning the bone over the paraclival carotid. Tumor was aspirated from the medial petrous apex toward the internal auditory canal and jugular tubercle. Bone was drilled to expose the dura, leaving a small bony cap to prevent CSF leak. No visible tumor remained; no transdural invasion was identified, but the tumor was clearly invasive.

The cavity was packed with TachoSil and Floseal for hemostasis. The Hadad flap was placed. The left hypoglossal nerve was not identified, likely still covered by bone.

### 2.4 Postoperative course

Postoperatively, the patient was free of new neurological deficits. The day before discharge we noticed a discrete palsy of the lower left corner of the mouth. On the first postoperative day, we performed a follow-up CT of the head, which showed the status post resection of most of the left middle and jugular portion of the clivus, sphenoid body, sphenoid sinus, left petrous apex, and ethmoid bone without significant hematoma. A 7 mm round hyperdense mass was described in the left pontocelebellar resection cavity, which continued from the emptied posterolateral portion of the resection cavity, suspected to be a small portion of surgical material with hemorrhagic contents.

### 2.5 Histopathologic examination

**Figure 3:**
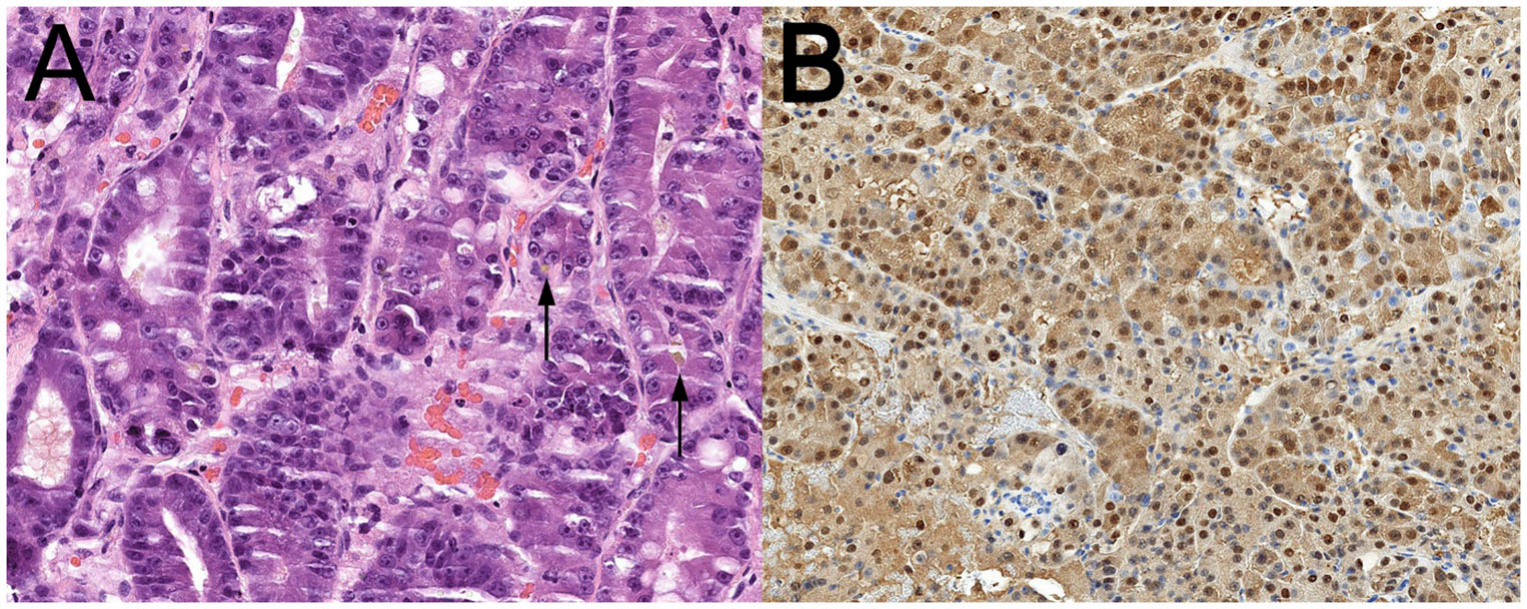
**(A)** Conventional HE-stained specimen slide reveals a solid, acinar and glandular growth of malignant tumor cells with abundant amphiphilic cytoplasm, vesicular nuclei and prominent nucleoli. Foci of brown bile pigment secretion are present (arrows). **(B)** Diffuse cytoplasmic and nuclear positivity of tumor cells for hepatocytic differentiation marker Arginase-1 by immunohistochemistry.

Conventional histopathologic examination on formalin-fixed paraffin-embedded tumor sample confirmed the frozen section impression, revealing a moderately differentiated adenocarcinoma with focal bile secretion (**Figure 3A**). Thus, targeted immunophenotyping by immunohistochemistry was performed to confirm the morphologic impression of metastatic HCC. The tumor cells were expectedly diffusely positive for markers of hepatocytic differentiation, such as arginase-1 (**Figure 3B**) and HepPar antigen. Additionally, oncofetal protein Glypican 3 and cytokeratin 18 were positive in keeping with the diagnostic idea of HCC.

### 2.6 Blood work-up

After receiving the report, blood is drawn for liver function tests, which show a highly elevated alpha-feto protein (AFP) levels of 1644.5 kU/L (ref. <6.2). AST (1.46 mckat/L, ref. <0.52), ALT (1.16 mckat/L, ref. <0.57) and gamma-GT (5.15 mckat/L, ref. <0.63) were also mildly elevated. All viral markers were negative.

### 2.7 Imaging studies reevaluation

**Figure 4:**
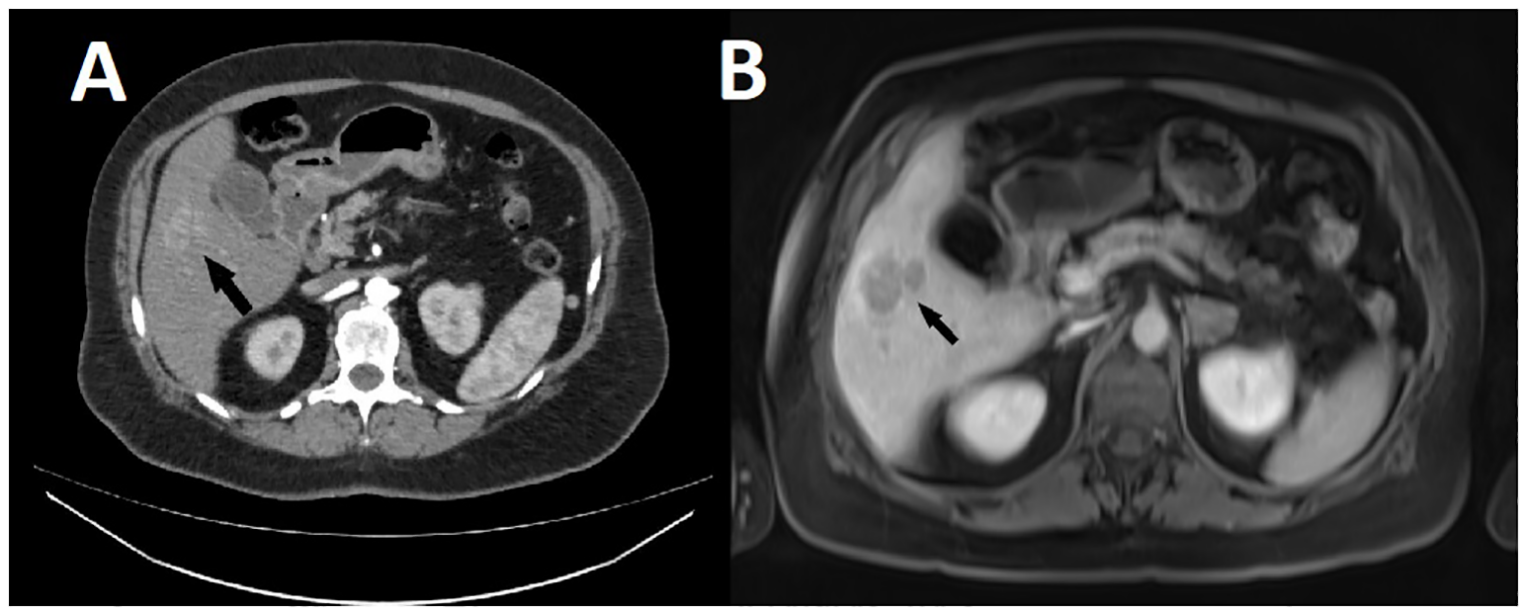
**(A)** CT scan of the abdomen performed 3 weeks before surgery for the clival tumor, showing discrete accumulation of contrast in the 5th segment of the liver (arrow). **(B)** MR scan of the liver performed 3 weeks after surgery, showing the same lesion (arrow), which correlated with the pathohistological findings and was diagnosed as multifocal hepatocellular carcinoma.

As already mentioned, she underwent an abdominal CT with contrast medium before the operation, in which the radiologist described no pathological changes in the liver (**Figure 4A**). After we received the pathohistologic report, we consulted the radiologists again to review the examination. After a thorough re-examination of the liver in correlation with pathohistologic findings, they reported that there might be a discrete accumulation of contrast in the 5th segment of the liver and recommended an MRI of the liver.

### 2.8 Follow-up and outcome

The patient recovered well from surgery and was able to go home on day 10, so she underwent outpatient imaging of the liver 3 weeks after surgery. Here they describe diffuse, slightly hyperintense lesions in the parenchyma on the T2 sequence, showing diffusion restriction, hypervascular in the arterial phase, larger and more numerous in the right lobe, the largest in segment 5 near the gallbladder, 3.6 cm in size, with contrast washout in the venous phase and a suggestive capsule. The others are mostly less than 1.2 cm in size, which makes their identification difficult, and are hypointense in the hepatobiliary phase. According to the MR features in correlation with the pathohistologic findings, it is most likely a multifocal hepatocellular carcinoma (**Figure 4B**). In addition, a 2.2 cm large lesion with diffusion restriction is newly discovered in the left adrenal gland, which becomes increasingly discolored after administration of contrast medium, probably a metastasis.

**Figure 5:**
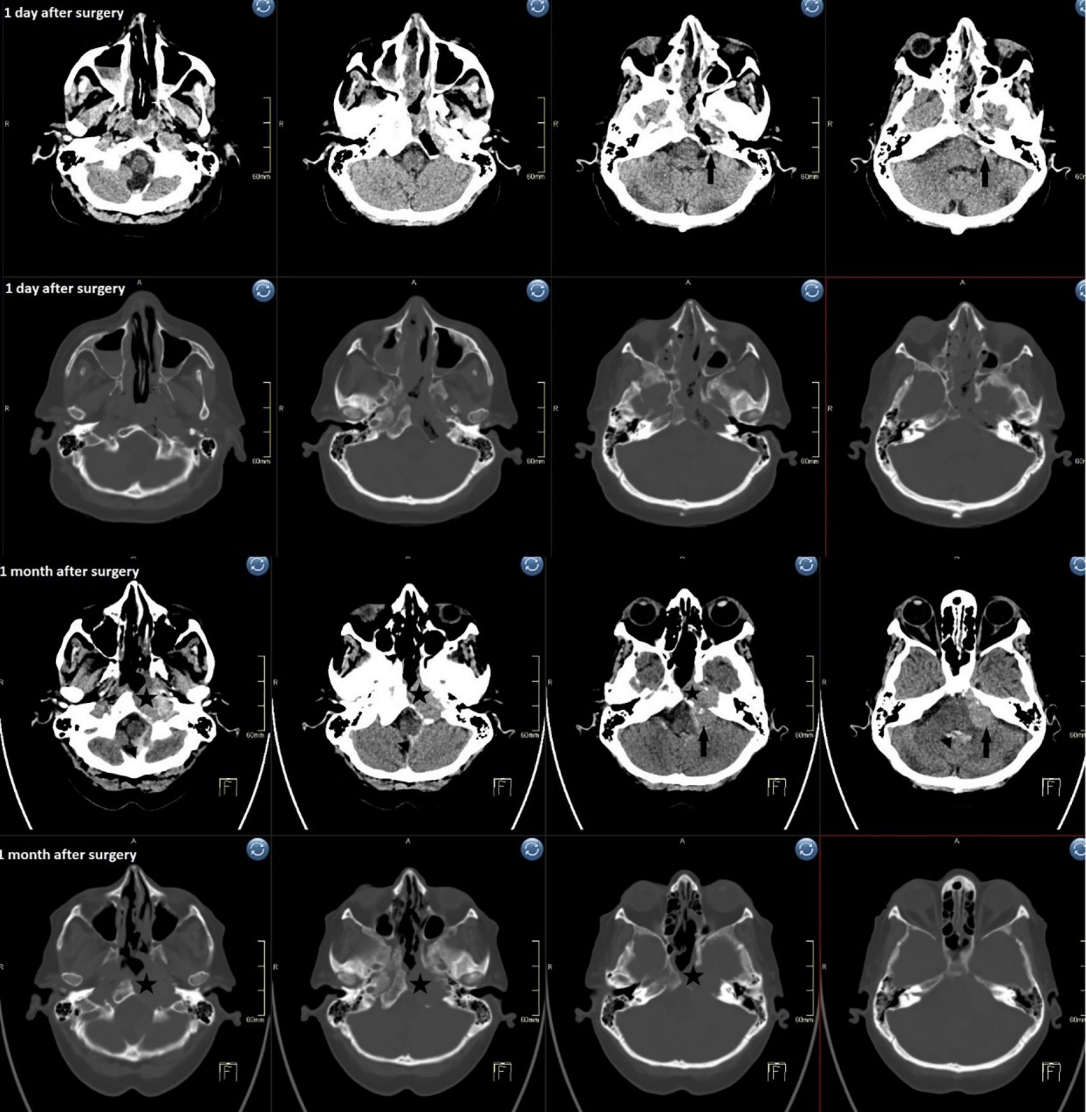
Postoperative CT 1 day (1st and 2nd row) and 1 month (3rd and 4th row) after surgery shows rapid regrowth of the tumor, especially the 7 mm intradural residual. The sudden deterioration started with headache, nausea and vomiting. CT showed SAH in the perimedullary cistern and blood in the fourth ventricle. *(star: tumor regrowth, arrow: 1st row small residual and 3rd row residual enlarged, triangle: haematocephalus in 4th ventricle)*.

The patient was presented to the hepatology consultation of the oncology institute, where the decision was made to perform palliative head irradiation. 1 month after surgery, she was re-examined in the neurology emergency department for nausea, vomiting, general weakness and a new onset of left facial nerve palsy, House-Brackmann grade 5, before presenting to the oncology institute. An urgent head CT is performed, which shows a well-circumscribed hyperdense mass in the resection cavity that is predominantly homogeneous and extends anterocaudally into the left cranial retropharyngeal space and pontocerebellary. The mass measures approximately 1.5 x 1.5 x 3 cm retropharyngeally and approximately 1.5 x 1.5 x 2.5 cm pontocerebellary (**Figure 5**). As mentioned above, the postoperative CT scan showed a 7 mm pontocerebellar area, which was clearly not a small portion of the surgical material with hemorrhagic contents, but a remnant of the tumor that had spread intracranially. During the transnasal endoscopic surgery, neither a defect in the clival opening nor a tumor remnant was found. A thin layer of fresh blood could be seen next to this mass, which also spread into the fourth ventricle, via the aqueduct into the third ventricle and via the foramina monro into the lateral ventricles. A layer of blood also spread caudally along the left cerebellar hemisphere and posteriorly into the foramen magnum. She was transferred back to our ward and treated symptomatically with fluid replacement, potassium and antiemetics. After a few days she stabilized and went to home care. After about 1 week, she was admitted to the Oncology Institute for palliative radiotherapy, where she received 5 fractions of 4 Gy each.

At the time of writing, she is in residential care. She is part of the Oncology Institute’s mobile palliative care team. She is generally debilitated, non-dependent, WHO performance status II - III with persistent severe paresis of cranial nerves VI, VII and XII.

## 3 Discussion

HCC is one of the most common causes of mortality in cancer patients. It is estimated that approximately 600–000 new cases are diagnosed annually (17). Most cases develop in cirrhotic liver parenchyma due to either alcoholism or infection with hepatitis C or B virus (18). It rarely occurs outside the context of cirrhosis as the first manifestation of liver disease. The exact percentage varies from author to author. Schütte et al. reported 14% HCC cases without prior cirrhosis (19), while Giannini et al. described only 1.7% (20). These cases are usually asymptomatic in the initial phase. By the time symptoms appear, the disease is so advanced that treatment is limited at the time of diagnosis. In our case, the first manifestation of the disease was cranial nerves VI and XII dysfunction due to compression of the clival metastasis. She did not report nausea, vomiting, general malaise, weight loss or inappetence.

Metastasis to the clivus is estimated to be at 0,02% of all intracranial tumors. Out of all clival metastases, the most common site of primary tumor was prostate, followed by gastrointestinal tract, lung and kidney (14). HCC metastases in the clivus are extremely rare, with only isolated cases described in the literature. A search in PubMed and Google Schoolar with the keywords “hepatocellular carcinoma”, “clivus” and “metastasis” yielded only 6 relevant hits (15, 21–25). Each of the cases described had either known cirrhosis or known HCC on a previously performed abdominal CT scan. Only the case of clivus metastasis described by Chaudhry et al. had no previously known liver pathology (21). Only after the biopsy of the clivus lesion, which turned out to be a metastasis of an HCC, was further imaging performed, in which an abdominal CT scan revealed primary disease. In our case, the routine diagnostic search for the primary tumor (the so-called CT triplet; neck, thorax and abdomen) was performed before surgery and did not reveal any suspicious lesions. Therefore, the pathohistological specimen was subsequently obtained for diagnostic purposes.

HCC can progress in 3 different ways: continuous spread to neighboring organs, hematogenous spread or lymphatic spread. If metastases are present in other organs or regional lymph nodes, surgical treatment is generally contraindicated (26). Palliative treatment is recommended. Conversely, our patient underwent surgery with an endonasal endoscopic approach as the primary disease was unknown. The aim of the surgery was mainly to narrow down the different diagnostic possibilities with intraoperative frozen section, to obtain adequate tissue for definitive diagnosis and, if possible, to maximize removal. Had the diagnosis of HCC been known prior to surgery, we would most likely have opted for confirmatory biopsy only. In the 2019 guidelines, only systemic treatment is recommended (27). Maximum resection does not improve the outcome, but can lead to serious consequences and worsen the quality of life of patients with an already poor prognosis.

In most cases, CT or MR imaging of the abdomen shows pathologic changes that are suspicious or diagnostic for HCC (28). In rare cases, these changes are not seen, e.g., when the tumor is small, has an atypical vascular pattern, is obscured by underlying liver disease, in rare subtypes of HCC, and due to imaging limitations. Since HCC is one of the more common malignancies, it may be useful to determine the alpha-feto protein (AFP) level in the patient’s blood if no primary disease is radiologically apparent. AFP is a biomarker that is often elevated in HCC patients (29). Values above 400 ng/ml are in principle already diagnostic for HCC. Our patient had a value of 1644.5 ng/mL. The sample was taken when the diagnosis was already known. If the AFP level had been determined earlier, major surgery and the risk of postoperative complications could have been avoided. On the other hand, AFP levels may have been low and of no diagnostic value at the start of treatment. Also, abdominal MR imaging was performed about 2 months after the abdominal CT scan and was merely suggestive of HCC in the context of definitive histopathologic diagnosis but not per se diagnostic. Perhaps the primary disease was still at such an early stage at the time of the preoperative abdominal CT scan that even an MRI would not have shown significant pathologic lesions at that time. Within 2 months of diagnosis, however, the disease had progressed rapidly, as HCC has potential for aggressive behavior. The extremely rapid local recurrence and progression of neurological symptoms after surgery (facial paresis) is remarkable.

## 4 Strengths and limitations

The major strength of this article is the publication of a case of HCC metastasis in the clivus in a patient with no known liver disease despite extensive preoperative imaging. To our knowledge, this is the first such case in the literature. This article emphasizes the importance of systematic management of the patient, searching for the underlying disease, and multidisciplinary collaboration between physicians of different specialties in selecting the optimal treatment. We believe it will provide readers with a starting point for the treatment of similar patients in the future.

Nevertheless, the article has its limitations. First, metastasis of HCC to the clivus is extremely rare. Only individual case reports are described. For this reason, it is not possible to publish a larger study on this pathology, on the basis of which the optimal treatment, the likelihood of complications and the results could be analyzed in more detail. In addition, the primary disease in patients with HCC metastasis in the clivus may be due to various chronic liver diseases such as viral hepatitis and alcoholism, but it may also occur outside the context of cirrhotic liver disease. This heterogeneity further complicates the possibility of a more precise analysis of the course of the disease.

## 5 Conclusion

Clival metastases of HCC are extremely rare. In the case of a radiologically suspected clival metastasis, it is important to reliably and promptly identify the primary cancer. If the imaging studies are inconclusive, it is reasonable to evaluate blood tumor marker levels. This can accelerate the time to diagnosis and avoid unnecessary surgery. If none of the studies are conclusive, the pathohistological tissue specimen evaluation remains a diagnostic gold standard. Nowadays, the surgical approach of choice for clival lesions is transnasal endoscopic. In some cases, intraoperative frozen section analysis can prevent overresection of unresectable tumors and prevent catastrophic complications, which allows faster postoperative recovery. Nevertheless, the treatment of metastatic clival tumors, including clival HCC metastasis, remains palliative, which maintains the patient’s quality of life.

## Data Availability

The raw data supporting the conclusions of this article will be made available by the authors, without undue reservation.
